# The underlying mechanisms of the persuasiveness of different types of satirical news messages

**DOI:** 10.1080/0163853X.2024.2381407

**Published:** 2024-08-20

**Authors:** Ellen Droog, Christian Burgers

**Affiliations:** a Department of Communication Science, Vrije Universiteit Amsterdam; b Amsterdam School of Communication Research (ASCoR), University of Amsterdam

## Abstract

Research into the persuasiveness of satirical news has found mixed results. Two possible explanations lie in the lack of clarity about mechanisms underlying the influence of consuming different types of satirical content. In six experiments (*N_total_* = 3,139), we investigated how (different types of) humorous versus nonhumorous (satirical) messages influenced recipients’ cognitive, emotional, and excitative responses and how these responses in turn influenced their attitudes. Results show that attitudes were influenced through recipients’ cognitive and emotional reactions to the stimuli but in opposite directions. This suppressed an overall effect on attitudes: Consuming humorous satirical messages led to more message-agreement because the messages were more humorous, and recipients felt less angry, while this consumption led to less message-agreement because the messages were discounted more, and recipients felt less worried. Our results highlight the importance of distinguishing between different types of satirical news content (humorous vs. nonhumorous) when studying satire’s persuasiveness.

## Introduction

Satirical news shows such as *the Daily Show* or *Last Week Tonight* are known for their discursive blends of entertaining, informative, and opinionative elements (Baym, 2005). Many scholars argue that such satirical news shows can be a powerful tool to persuade audiences (Holbert, 2013; Waisanen, 2018). However, previous studies have found no strong and consistent evidence for satire’s ability to directly influence attitudes toward social issues (Boukes et al., 2015; Burgers & Brugman, 2021). In this article, we aim to advance our understanding of satire’s persuasiveness by empirically testing two possible and complementary explanations for these inconsistent findings in previous literature.

The first explanation builds on the observation that many satire studies have primarily focused on the direct effects of satirical news consumption on viewers’ attitudes, without considering the indirect ways through which satire may or may not be persuasive (Boukes et al., 2015). The Differential Susceptibility to Media Effects Model (DSMM; Valkenburg & Peter, 2013) proposes that three core mediator types underlie the relationship between media consumption and media effects: cognitive responses (e.g., attention or cognitive effort), emotional responses (e.g., happiness or sadness), and excitative responses (e.g., arousal levels). Satire studies have shown that these different mediator types sometimes counteract each other’s influence on persuasion (Boukes et al., 2015; Droog et al., 2023). For example, satirical news can impede persuasion through higher levels of perceived funniness, causing recipients to perceive the information as less serious, making them more likely to counter-argue the information (Boukes et al., 2015). Simultaneously, satirical news can enhance persuasion by fostering greater absorption into the satire, demanding significant cognitive resources from recipients, making it more challenging for them to resist the presented information (Boukes et al., 2015).

However, the literature does not only indicate that different mediator types may have differential effects (e.g., cognitive vs. emotional responses) but also suggests that the same mediator type may work in different ways. A specific mediator type namely represents various underlying mechanisms; emotional responses as a mediator type for example, consist of both positive and negative emotions. Research shows that satirical news consumption elicits positive emotions which consequently enhance persuasion, while simultaneously also eliciting negative emotions which in turn impede persuasion (Droog et al., 2023). As a result, the cumulative direct effect of satire on attitudes remains inconclusive. Therefore, the first novel contribution of our study is that we want to create a better understanding of the specific underlying mediating mechanisms *through* which satirical news consumption may or may not be persuasive. We do this by adopting the framework of the DSMM and investigating how satirical news consumption influences recipients’ cognitive, emotional, and excitative responses, and how these response states in turn, impact persuasion.

Building on this indirect-effects model of satirical news, our second explanation is derived from the fact that most studies into satire’s persuasiveness have focused on intergenre differences between satirical news and other genres (e.g., regular news; Burgers & Brugman, 2021). However, the genre of satirical news is nonmonolithic and consists of various types of satirical content (Holbert et al., 2011). The satire literature for example, often differentiates between Horatian (a softer form) and Juvenalian forms of satire (a harsher form; Holbert et al., 2011; LaMarre et al., 2014). The DSMM argues that such content-related differences can have a differential impact on recipients’ cognitive, emotional, and excitative responses (Valkenburg & Peter, 2013). This is also evidenced by research that shows that Horatian satire is for example, taken less seriously than Juvenalian satire, which in turn impedes the persuasiveness of the satirical message (LaMarre et al., 2014). It is therefore crucial to also focus on the influence of differences in content-related features *within* the genre of satirical news (also known as intragenre differences). Therefore, the second novel contribution of our study is that we create a better understanding of which specific types of satirical news content lead to which specific types of responses in recipients.

In a series of six experiments, we therefore investigated how the consumption of different satirical messages influences recipients’ cognitive, emotional and excitative responses, and how these responses in turn influence recipients’ message-congruent attitudes. Experiments 1 to 3 focus on various cognitive responses to these different types of messages (i.e., message discounting and resource allocation), while Experiments 4 to 6 deal with various emotional (i.e., happiness, hopefulness, sadness, and worries) and excitative (arousal) responses.

### Content-related features of satirical news

Satirical news is a hybrid genre that offers humorous news updates containing critiques of political, economic, or social affairs (Baym, 2005; Tandoc et al., 2018). However, research investigating content-related differences in satirical news demonstrates that satirical news consists of a mixture of fun and facts (Fox, 2018). In satire, the use of humor is often interspersed with serious information delivered in a nonhumorous manner (Fox, 2018). Thus, humor is not the only defining characteristic of satire, and satirists can also criticize and/or explain political, economic, or societal issues without being humorous. Research from the area of humor studies shows that consuming humorous (vs. nonhumorous) messages elicits various cognitive, emotional, and excitative responses that can simultaneously impede as well as enhance persuasion (Eisend, 2009, 2011). This means that the consumption of both the humorous and nonhumorous satirical information in a satirical news episode might also simultaneously evoke opposing underlying mediating processes that may prevent an overall persuasive effect of satirical news consumption. In this study, we therefore investigate the differential effect of humorous versus nonhumorous (satirical) messages on recipients’ cognitive (Experiments 1–3) and emotional and excitative responses (Experiments 4–6).

Furthermore, when looking even more closely at satirical news’ content-related features, there are numerous ways through which these humorous and critical news updates can be delivered, one of which is the use of figurative language (Droog et al., 2020; Young, 2019; Young et al., 2019). This study, therefore, also focuses on investigating the distinct effects of two specific forms of figurative language that are often used in satirical news to entertain, inform, and persuade audiences: similes and hyperboles. However, despite their importance, these rhetorical devices have received relatively limited empirical attention within the realm of satire research (Droog et al., 2020; Young, 2019; Young et al., 2019).

Similes are a form of direct metaphors (e.g., “Joe Biden is like a comfortable pair of shoes”; Rubin, 2019) which are defined as cross-domain mappings in which information from a source domain (clothes) is explicitly mapped onto a target domain (politics; Lakoff & Johnson, 1980/2003). In metaphor research, a distinction is often made between direct and indirect metaphors (Steen et al., 2010). Direct metaphors (e.g., similes or nonliteral analogies) are expressions for which recipients must compare two different concepts (politics and clothes) to be able to understand what they have in common (e.g., they make you both feel at ease). By contrast, for indirect metaphorical expressions (e.g., “Joe Biden *attacks* Donald Trump in final presidential debate”; NBC News, 2020), the cross-domain mapping is implied rather than explicitly expressed in language. Therefore, understanding indirect metaphors does not occur through a process of comparison, but rather through a process of categorization (Gentner & Bowdle, 2001). Moreover, recent research shows that direct metaphors such as similes abound in satirical news shows and are often used by satirists to humorously explain and/or criticize current affairs (Droog & Burgers, 2023; Droog et al., 2020).

Hyperboles (e.g., “Joe Biden is so old that when he was in school there was no history class”) are defined as expressions that are more extreme than justified, given their ontological referent (Burgers et al., 2016). When hyperboles are used humorously, they can be considered as a form of scalar humor. This is a type of humor that is based on recipients’ assumptions about the probable values of a conceptual scale (Bergen & Binsted, 2004). To understand this type of humor, recipients must reconcile the claim in the first clause of the hyperbole (Joe Biden is so old…) with the alleged result of his age depicted in the second clause of the hyperbole (… when he was in school there was no history class). Hyperboles are inherently evaluative (Burgers et al., 2016) and are therefore used by satirists to humorously criticize current affairs (Young, 2019).

Although similes and hyperboles are both forms of figurative language, they differ in their structure and processing demand and can therefore also differentially impact the processing of its figurative information (Rubio-Fernández et al., 2015; Toncar & Munch, 2003). We therefore also investigate if humorous satirical messages including similes versus hyperboles elicit different cognitive (Experiments 1–3) and emotional and excitative responses in recipients (Experiments 4–6).

## Experiments 1–3

### Cognitive responses and message-congruent attitudes

The literature distinguishes various types of cognitive responses (Valkenburg & Peter, 2013). Most theoretical explanations for why satirical news consumption can have persuasive effects from an intergenre perspective are grounded in two cognitive processing principles: the message-discounting and resource-allocation principles (Nabi et al., 2007; Young, 2008). The message-discounting principle assumes that recipients discount satirical stimuli as “just a joke”. This discounting cue reduces recipients’ motivation to critically process the political arguments in the satirical stimuli, because the stimuli is just a joke and therefore not worthy of consideration (compared to nonhumorous stimuli; Nabi et al., 2007). The resource-allocation principle assumes that understanding satirical stimuli requires a substantive amount of cognitive effort. This reduces recipients’ ability to critically process the political arguments in the satirical stimuli, because recipients have few cognitive resources left to think about them (compared to nonhumorous stimuli; Young, 2008).

In this intergenre perspective, the empirical evidence for the working of either one of these underlying cognitive processing principles is mixed (Polk et al., 2009). However, by adopting an intragenre perspective, we can more precisely investigate if and how the differential cognitive processing of the humorous and nonhumorous messages in satirical news can explain these inconsistent findings. This leads toward the following hypothesis:

*H1*: The consumption of a humorous satirical message will result in *(a)* more message discounting and *(b)* more resource allocation than the consumption of a nonhumorous (satirical) message.

Some scholars argue that the type of cognitive processing depends on the type of humorous satirical news content that is consumed (Holbert et al., 2011; LaMarre et al., 2014). According to these intragenre studies, the complexity and sense of urgency of a satirical joke determines whether the joke is processed through the message discounting or the resource allocation principle (Holbert et al., 2011; LaMarre et al., 2014). Because the use of humor in the form of a simile can also be seen as a more urgent and complex form of humor than humor in the form of a hyperbole (McQuarrie & Mick, 1996), these different types of humorous satirical messages might also induce different levels of message discounting and resource allocation.

First, a humorous simile can be used as a concise way to convey a set of arguments that are perceived as important by recipients, which might evoke a sense of urgency (LaMarre et al., 2014; Ottati & Renstrom, 2010; Toncar & Munch, 2003; Whaley & Holloway, 1997). Hyperbolical humor, by contrast, is probably less capable of conveying this sense of urgency because this type of humor provides more playful humorous commentary, without giving much credence to arguments (LaMarre et al., 2014; Young, 2019). This might make recipients likelier to dismiss the message as “just a joke”. This means that consuming a humorous hyperbole might cue more message discounting than consuming a humorous simile (Nabi et al., 2007).

Second, understanding similes is cognitively taxing because recipients must compare two different concepts to each other (Bowdle & Gentner, 2005). This means that understanding humorous similes implies a multidimensional qualitative shift away from the encoded meaning of the target of the simile (Rubio-Fernández et al., 2015). In contrast, understanding humorous hyperboles requires less cognitive processing demand than humorous similes since the intended meaning of a hyperbole is more explicit. This is because understanding a hyperbole implies a shift of magnitude along a qualitative or quantitative scale that is inherently linked to the encoded meaning of the hyperbole target (Rubio-Fernández et al., 2015). Therefore, consuming humorous similes might cue more resource allocation than consuming humorous hyperboles (Young, 2008).

*RQ1*: To what extent does the consumption of different types of humorous satirical messages (similes vs. hyperboles) result in different levels of message discounting and resource allocation?

Both the message discounting and the resource allocation principles predict that consuming humorous satirical messages compared to a nonhumorous (satirical) message should reduce counter-argumentation to the message (although in varying levels; LaMarre et al., 2014), either through a reduced motivation (message discounting) or reduced ability (resource allocation) to do so (Polk et al., 2009). In turn, the less recipients counter-argue the humorous satirical message, the more they will agree with the message (Nabi et al., 2007; Young, 2008). We therefore expect the following:

*H2*: Recipients’ levels of *(a)* message discounting and *(b)* resource allocation function as mediators in the relationship between the consumption of humorous versus nonhumorous (satirical) messages and message-congruent attitudes.

### Methods

#### Design

We conducted three experiments using three different societal issues. All experiments used the same procedure and instrumentation but varied slightly in design.^1^ Experiment 1 contained a unifactorial between-subjects design with three conditions (type of satirical message: humorous simile vs. humorous hyperbole vs. a nonhumorous satirical message). In Experiments 2 and 3, we added a fourth (control) condition (a nonhumorous regular news message) to investigate the differential impact of consuming nonhumorous messages with either a satirical or regular news source. This allowed us to check if satirical news’ impact solely depends on content-related aspects like humor, rather than its satirical nature.

#### Participants

US participants were recruited in January and February 2022 through the online data panel Prolific and were compensated with $0.83 for their participation (5 minutes). Sample sizes for the experiments were determined a priori using G*Power 3.1. To test our hypotheses and research questions with sufficient power (0.80), based on a small effect size of *f* = 0.15 (Walter et al., 2018), we required a total sample of 432 for Experiment 1 and 492 for Experiments 2 and 3. To accommodate a ±10% dropout rate, we aimed for 500 participants for Experiment 1 and 550 participants for Experiments 2 and 3. Participants could participate in only one of the experiments reported in this article. See Table 1 for the demographic characteristics of each experiment.

**Table 1.** Demographic characteristics of participants across all experiments.

Demographic characteristics	Experiment 1 (*N* = 486)	Experiment 2 (*N* = 544)	Experiment 3 (*N* = 541)	Experiment 4 (*N* = 489)	Experiment 5 (*N* = 543)	Experiment 6 (*N* = 536)
Gender						
Female	327 (67.3%)	361 (66.4%)	325 (60.1%)	324 (66.3%)	350 (64.5%)	331 (61.8%)
Male	148 (30.5%)	170 (31.3%)	204 (37.7%)	157 (32.1%)	186 (34.3%)	193 (36.0%)
Other	11 (2.2%)	13 (2.4%)	12 (2.3%)	8 (1.6%)	7 (1.3%)	12 (2.2%)
Age						
Mean (SD)	33.49 (11.29)	34.71 (13.36)	35.74 (12.96)	33.45 (11.95)	32.88 (11.45)	35.79 (13.76)
Range	18–78	18–81	18–92	18–74	18–75	18–79
Educational level						
Less than high school	2 (0.4%)	4 (0.7%)	6 (1.1%)	6 (1.2%)	8 (1.5%)	6 (1.1%)
High school	54 (11.1%)	52 (9.6%)	62 (11.5%)	48 (9.8%)	62 (11.4%)	73 (13.6%)
College but no degree	122 (25.1%)	159 (29.2%)	116 (21.4%)	111 (22.7%)	134 (24.7%)	116 (21.6%)
Associate degree	38 (7.8%)	43 (7.9%)	49 (9.1%)	39 (8.0%)	51 (9.4%)	53 (9.9%)
Bachelor’s degree	195 (40.1%)	200 (36.8%)	216 (39.9%)	206 (42.1%)	200 (36.8%)	215 (40.1%)
Master’s degree	51 (10.5%)	73 (13.4%)	65 (12.0%)	57 (11.7%)	69 (12.7%)	53 (9.9%)
Professional degree	9 (1.9%)	6 (1.1%)	19 (3.5%)	11 (2.2%)	7 (1.3%)	9 (1.7%)
Doctorate degree	12 (2.5%)	6 (1.1%)	6 (1.1%)	11 (2.2%)	11 (2.0%)	10 (1.9%)
Other	3 (0.6%)	1 (0.2%)	2 (0.4%)	0 (0.0%)	1 (0.2%)	1 (0.2%)
Political Ideology						
Mean (SD)	2.97 (1.67)	2.96 (1.64)	3.10 (1.64)	2.95 (1.68)	2.86 (1.63)	3.07 (1.75)

Political ideology was measured on a seven-point scale ranging from 1 = very liberal to 7 = very conservative.

#### Materials

The stimulus materials consisted of three issues that differed in geographical focus to increase external validity and generalizability of our findings: a global issue (climate change), a national issue (student loan debt), and a foreign issue (Brexit). Participants were presented with a short (fictitious) transcript of a fragment from either the satirical news show the *Daily Show with Trevor Noah* or NBC’s regular news show *Nightly News with Lester Holt*. Transcripts were created for research purposes but were based on actual American (satirical) news discourse. All transcripts began with an opening statement introducing the issue of the segment (e.g., “Trevor Noah/Lester Holt: Tonight, we are talking about student loan debt”). This opening statement was followed by some general information about the (severity of the) issue (e.g., “Student loans are affecting millions of people, many of them just out of college…”). The transcripts ended with our manipulation of the (satirical) message types. Participants either saw a satirical humorous message containing a simile, a satirical humorous message containing a hyperbole, a nonhumorous satirical message, or a nonhumorous regular news message (control condition), which were all closely matched so that they contained the same evaluative statement about the issue.

All humorous satirical messages containing a simile were written in the analogical form of “A is like B” and contained a negative evaluation by explaining why the target issue “A” is like the negative source “B” (Droog et al., 2020; e.g., “Student loan debt is like the new herpes. Almost everybody has it, it stays with you your whole life, and eventually you’re gonna have to tell your partner about it”). The humorous satirical messages containing a hyperbole, included a qualitative exaggeration of the issue described in the general information (e.g., “Most student loans take so long to repay, that many of you will still be paying off your loans after you have been in heaven for over a thousand years”). The nonhumorous messages contained the same evaluative messages as the humorous messages, but were nonhumorous and nonfigurative (e.g., “Most student loans take so long to repay that many of you will still be paying of your loans well into middle age”). The nonhumorous regular news messages were the same as the nonhumorous satirical messages but contained a different source cue (i.e., NBC’s Lester Holt). See Appendix A on the Open Science Framework (https://edu.nl/a6hh3) for all transcripts.

#### Procedure

Data were collected online through Qualtrics. Procedure and instrumentation were roughly equal across the experiments.^2^ After providing consent, participants were randomly shown one of the three (Experiment 1) or four (Experiments 2 and 3) experimental conditions. After reading the transcript, participants completed a questionnaire including an attention check and items measuring message discounting, resource allocation, perceived humor, source liking, and message-congruent attitudes. Finally, we tapped into several demographic variables; age, gender, educational level, political ideology and regular/satirical news consumption. After completing all questions, participants were thanked and debriefed.

#### Measures

##### Message discounting

Message discounting was measured by asking participants on a seven-point Likert scale from (1 = completely disagree to 7 = completely agree) to what extent they agreed with the following four items (Nabi et al., 2007): “The host was just joking”, “The message in the transcript was intended more to entertain than to persuade”, “The host was serious about advancing his views in the transcript (reversed)”, and “It would be easy to dismiss the message in the transcript as simply a joke” (Experiment 1: *M* = 2.82, *SD* = 1.25, α = .82; Experiment 2: *M* = 2.56, *SD* = 1.17, α = .81; Experiment 3: *M* = 3.09, *SD* = 1.26, α = .82).

##### Resource allocation

Resource allocation was measured by asking participants on a seven-point Likert scale from (1 = completely disagree to 7 = completely agree) to what extent they agreed with the following three items (LaMarre et al., 2014; Young, 2008): “I found it difficult to think about the political message in the transcript”, “I was able to pay close attention to the political message in the transcript (reversed)”, and “it was easy to understand the political message in the transcript (reversed)” (Experiment 1: *M* = 2.21, *SD* = 1.16, α = .86; Experiment 2: *M* = 2.10, *SD* = 1.01, α = .88; Experiment 3: *M* = 2.30, *SD* = 1.06, α = .84).

##### Message-congruent attitudes

Message-congruent attitudes were measured by asking participants to indicate on slider scales (0–100) to what extent they believed that “climate change is a problem for the planet” (Experiment 1: *M* = 81.20, *SD* = 21.82), “student loan debt is a problem for people in the United States” (Experiment 2: *M* = 65.90, *SD* = 20.53), and “the consequences of Brexit are problematic for Britain” (Experiment 3: *M* = 69.73, *SD* = 20.88).

##### Perceived humor

As a manipulation check, we asked participants to what extent they agreed with the following statement: “I think the transcript was humorous” (Experiment 1: *M* = 3.86, *SD* = 1.91; Experiment 2: *M* = 3.40, *SD* = 2.05; Experiment 3: *M* = 3.67, *SD* = 1.89).

#### Data analysis

To test *H1*, we conducted independent sample *t*-tests on the humorous versus nonhumorous (satirical) messages.^3^ To create these two conditions, we collapsed the humorous similes and hyperboles into a “humorous message” condition and the nonhumorous satirical and nonhumorous regular news conditions into a “nonhumorous” message condition. To answer *RQ1*, we conducted one-way ANOVAs on all the different types of (satirical) messages, with Bonferroni multiple comparisons tests. To investigate *H2*, we carried out mediation analyses on the humorous versus nonhumorous (satirical) messages with multiple mediators using the Process macro v3.4 for SPSS statistics (Hayes, 2017; model 4; 5,000 bootstap samples). To generalize our findings across experiments, we conducted multiple mini meta-analyses on the results (Goh et al., 2016). For the direct effects, we used the Major package (Hamilton, 2018) in Jamovi (version 2.2.5). The analyses were carried out using the correlation coefficient as the outcome measure and fixed-effects models were fitted to the data. For the indirect effects, we used meta-analytic structural equation modeling analyses (MASEM; Jak, 2015) with the metaSEM package (Cheung, 2015) in R (4.0.5). In this article, we only report the results of these mini meta-analyses to support the hypotheses. The results of all individual analyses are reported in Appendix D, while the data and data analyses are reported in Appendix E.

### Results

#### Manipulation checks

Table 2 shows the differences in participants’ levels of perceived humor between all different (satirical) messages (statistical results of the analyses are presented in Appendix F). The results of our mini meta-analysis show that consuming humorous versus nonhumorous (satirical) messages resulted in higher levels of perceived humor (Table 3). Therefore, the manipulations were successful. Furthermore, in addition to serving as a manipulation check, studies show that perceived humor can also play an important mediating role in the relationship between satire consumption and recipients’ attitudes. While some studies find positive relationships between perceived humor and attitudes (e.g., Hsieh et al., 2012), others find negative effects (e.g., Boukes et al., 2015). However, perceived humor cannot be easily placed into only one of the underlying mechanisms of the DSMM, because humor includes both cognitive (i.e., attracts attention), emotional (initiates affective responses), and excitative elements (increases arousal). Therefore, we also included perceived humor as a separate variable in our mediation analyses.

**Table 2.** Means and (SDs) of dependent variables across conditions of Experiments 1–3.

	Condition	*n*	Perceived Humor	Message Discounting	Resource Allocation	Attitudes
Experiment 1 Climate change (*N* = 486)	S	164	4.85 (1.52)^a^	3.06 (1.18)^a^	2.25 (1.18)^a^	82.05(19.08)^a^
	H	159	4.47 (1.63)^a^	3.22 (1.26)^a^	2.18 (1.13)^a^	83.37 (20.36)^a^
	NHS	163	2.25 (1.42)^b^	2.19 (1.05)^b^	2.19 (1.17)^a^	78.23 (25.31)^a^
Experiment 2 Student loan debt (*N* = 544)	S	137	5.37 (1.35)^d^	3.30 (1.19)^d^	1.94 (0.81)^d^	68.09 (19.26)^d^
	H	134	4.45 (1.69)^e^	2.95 (1.17)^e^	1.98 (0.87)^d^	65.35 (21.10)^d^
	NHS	137	2.29 (1.32)^f^	2.16 (0.97)^f^	2.08 (1.02)^de^	65.61 (21.37)^d^
	NHR	136	1.49 (0.75)^g^	1.85 (0.64)^f^	2.39 (1.23)^e^	64.53 (20.39)^d^
Experiment 3 Brexit (*N* = 541)	S	133	4.66 (1.66)^h^	3.46 (1.09)^h^	2.25 (1.07)^h^	69.56 (20.00)^h^
	H	137	4.80 (1.61)^h^	3.85 (1.23)^i^	2.40 (1.10)^h^	68.46 (21.61)^h^
	NHS	139	3.01 (1.62)^i^	2.79 (1.15)^j^	2.22 (1.02)^h^	70.22 (21.38)^h^
	NHR	132	2.21 (1.28)^j^	2.24 (0.88)^k^	2.34 (1.05)^h^	70.70 (20.62)^h^

S = humorous satirical message containing a simile; H = humorous satirical message containing a hyperbole; NHS = nonhumorous satirical message; NHR = nonhumorous regular news message. Expect for message-congruent attitudes, which was measured on a scale from 0 to 100, all variables were measured on seven-point scales. Different superscripts in the same column of each experiment indicate significant differences of at least *p* < .05. Each experiment uses its own set of superscripts.

**Table 3.** Results of mini meta-analyses total effects.

				95% C.I.				
IV	DV	*r*	SE	Lower	Upper	*df*	*t*	*p*
**Experiments 1–3**								
Humorous vs. nonhumorous	Message perceptions							
	Perceived humor	0.67	0.01	0.61	0.73	2	47.17	<.001
	Cognitive responses							
	Message discounting	0.44	0.02	0.35	0.53	2	21.70	.002
	Resource allocation	−0.04	0.03	−0.15	0.07	2	−1.58	.256
	Persuasion							
	Message-congruent attitudes	0.03	0.03	−0.07	0.14	2	1.37	.303
**Experiments 4–6**								
Humorous vs. nonhumorous	Message perceptions							
	Perceived humor	0.60	0.02	0.53	0.67	2	36.48	<.001
	Emotional responses							
	Hopefulness	0.03	0.03	−0.08	0.13	2	1.00	.424
	Happiness	0.21	0.02	0.10	0.31	2	8.47	.014
	Anger	−0.16	0.02	−0.26	−0.05	2	−6.31	.024
	Worry	−0.12	0.02	−0.23	−0.02	2	−5.01	.038
	Excitative responses							
	Excitement	0.11	0.02	0.002	0.022	2	4.37	.049
	Persuasion							
	Message-congruent attitudes	−0.01	−.03	−0.12	0.10	2	−0.25	.824

#### Cognitive responses

In relation to *H1*, the results of our mini meta-analysis show that consuming humorous (vs. nonhumorous) messages resulted in higher levels of message discounting, but not in higher levels of resource allocation (see Table 3). Therefore, results support *H1a*, but not *H1b*.

Regarding *RQ1*, we found no differences in participants’ cognitive responses between the humorous similes and humorous hyperboles (see Table 2 and Table F1 in Appendix F). Overall, this means that there are no consistent differences in participants’ cognitive responses between the humorous similes and humorous hyperboles.

#### Message-congruent attitudes

The results of our mini meta-analysis show that consuming humorous (vs. nonhumorous) messages did not directly result in more message-congruent attitudes (Table 3). We also found no significant differences in message-congruent attitudes between all the different types of (satirical) messages (see Table 2 and Table F1 in Appendix F).

**Figure 1.**
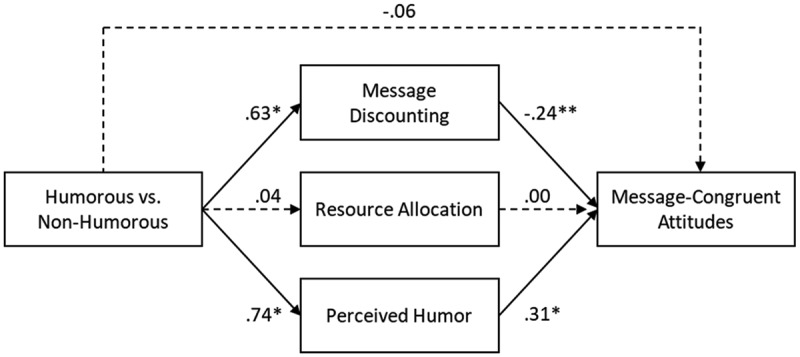
Meta-analysis of indirect effects Experiment 1–3 (cognitive responses). Standardized regression estimates are shown. Paths with continuous lines are significant and paths with dotted lines are not significant. *p < .05, **p < .001.

Regarding *H2*, the results of our mini meta-analysis show that the relationship between consuming humorous versus nonhumorous messages and message-congruent attitudes was mediated through both message discounting and perceived humor but not through resource allocation.^4^ Therefore, these results support *H2a* but do not support *H2b*. Consuming humorous (vs. nonhumorous) messages evoked two opposing underlying processes that might have suppressed an overall effect on attitudes: on the one hand, consuming humorous (vs. nonhumorous) messages led to more perceived funniness of the message, which resulted in more message-congruent attitudes. On the other hand, consuming humorous (vs. nonhumorous) messages led to more message discounting, which resulted in less message agreement (Table 4 and Figure 1).

**Table 4.** Results of mini meta-analyses indirect effects.

			95% LBCI	
		Estimate	Lower	Upper
**Experiment 1–3: Cognitive Responses**				
Indirect	C → MD → MCA	−0.15	−0.20	−0.11
	C → RA → MCA	0.00	−0.002	0.003
	C → PH → MCA	0.23	0.17	0.30
Direct	C → MCA	−0.06	−0.17	0.05
**Experiment 4–6: Emotional and Excitative Responses**				
Indirect	C → H → MCA	0.03	−0.09	0.14
	C → A → MCA	0.06	0.02	0.09
	C → W → MCA	−0.18	−0.22	−0.15
	C → E → MCA	−0.006	−0.06	0.05
	C → PH → MCA	0.16	0.10	0.22
Direct	C → MCA	−0.13	−0.36	0.11

C = humorous vs. nonhumorous (satirical) messages; MD = message discounting; RA = resource allocation; PH = perceived humor; MCA = message-congruent attitudes H = happiness; A = anger; W = worry; E = excitement.

### Discussion

In line with our expectations (*H1a*), we found that recipients who consumed humorous (vs. nonhumorous) messages perceived these messages to be less serious, while, contrary to our expectations (*H1b*), they were not more difficult to understand. This indicates that humorous (vs. nonhumorous) satirical messages may be more likely to be cognitively processed through the message discounting rather than the resource allocation principle (Nabi et al., 2007; Young, 2008).

Recipients’ message-congruent attitudes were influenced through two underlying processes: perceived humor and message discounting. Consuming humorous versus nonhumorous (satirical) messages led to more message-congruent attitudes because recipients perceived the messages as more humorous, while at the same time this consumption led to less message-congruent attitudes because recipients discounted these messages more (*H2a*), thereby suppressing an overall effect on recipients’ message-congruent attitudes. This message discounting finding is contrary to our expectations, in which we argued that message discounting of humorous messages would lead to less motivation to counter argue the messages, and thus would enhance message-agreement (Polk et al., 2009). In contrast, this finding is more in line with the original predictions of the message discounting principle (Nabi et al., 2007), which argues that discounting a humorous message as just a joke increases counter-arguing to the message and thus undermines its persuasive effect (Nabi et al., 2007).

Moreover, our findings show no consistent differences in how different forms of humorous messages (similes vs. hyperboles) influence recipients’ levels of message discounting and resource allocation (*RQ1*). This means that the specific form through which humorous criticism in a message is delivered does not seem to influence how seriously recipients take this message, or how difficult they find the message to understand (Bowdle & Gentner, 2005; Rubio-Fernández et al., 2015; Young et al., 2019).

In Experiments 1 to 3, we focused on the cognitive responses to different types of (satirical) news messages. However, research shows that different types of media content can also impact more affective reactions such as emotional or excitative responses (Valkenburg & Peter, 2013). In Experiments 4 to 6, we will therefore focus on the differential impact of different types of (satirical) news messages on recipients’ emotional and excitative responses.

## Experiments 4 to 6

### Emotional and excitative responses and message-congruent attitudes

Over the past years, we have seen a growing interest in the emotional processing of satirical news (Droog et al., 2023). This research has often been characterized by the use of a discrete emotion perspective in which emotions are considered to be categorical emotional states that arise from a recipient’s appraisal of a media stimulus (Lazarus, 1991; Nabi, 2010). In this view, content-related aspects of a media stimulus can influence which or how strongly specific emotions are evoked (Nabi, 2010). Therefore, it is important to use such a discrete emotion perspective when investigating the intragenre differences in satirical news content, because different types of satirical messages may induce different appraisals and can thus differently impact recipients’ emotional responses (Droog et al., 2023).

From an intergenre perspective, the literature quite consistently shows that consuming satirical (vs. regular) news generates stronger positive emotional responses like mirth, enjoyment, happiness, or pleasure because of its entertaining nature (Burgers & Brugman, 2021; Peifer & Landreville, 2020). By contrast, the critical nature of satire can lead to appraisals that induce stronger negative emotional responses like anger, sadness, or worry than regular news (Chen et al., 2017; Droog et al., 2023; Lee & Kwak, 2014). From an intragenre perspective, consuming humorous (vs. nonhumorous) messages also evoke stronger positive emotions that result from resolving the incongruity in the message (Eisend, 2009, 2011). However, consuming such humorous (vs. nonhumorous) messages actually reduces recipients’ negative emotional responses to these messages (Eisend, 2009, 2011). This is because humor results in a reappraisal of the negative message, in that the message is being reevaluated as something that is less threatening (Lefcourt & Martin, 2012; Samson & Gross, 2012). This results in the following hypothesis:

*H3*: The consumption of a humorous satirical message will result in fewer negative feelings of *(a)* anger and *(b)* worry, and in more positive feelings of *(c)* happiness and *(d)* hopefulness than the consumption of a nonhumorous (satirical) message.

In the satire literature, recipients’ excitative responses to the consumption of satirical news have barely received any scholarly attention (Droog et al., 2023). One study that did investigate recipients’ levels of arousal showed that from an intergenre perspective, consuming satirical (vs. regular) news stimuli lowered viewers’ physiological arousal levels, while there were no differences in viewers’ self-reported levels of arousal (Droog et al., 2023). However, from an intragenre perspective, humorous (vs. nonhumorous) messages are shown to evoke higher levels of arousal (Walter et al., 2018). According to the arousal theory of humor (Berlyne, 1972), this is because humor contains properties such as novelty, surprise, complexity, ambiguity, and incongruity that requires mental effort to be resolved and therefore increases recipients’ arousal levels. This leads toward the following hypothesis:

*H4*: The consumption of a humorous satirical message will result in more feelings of excitement than the consumption of a nonhumorous (satirical) message.

So far, it remains unclear whether different types of humorous satirical messages can also differ in the kind, or the intensity of the emotional and excitative responses they elicit. However, if similes evoke a greater sense of urgency than hyperboles (Ottati & Renstrom, 2010; Toncar & Munch, 2003; Young, 2019), we could argue that recipients might perceive humorous similes as being more significant to their personal goals than humorous hyperboles (Lazarus, 1991).

To illustrate, if a humorous hyperbolic message about the consequences of a particular societal issue does not induce a sense of urgency (compared to a humorous message containing a simile), this hyperbolical message will likely not really threaten recipients’ personal goals and desires, because the recipient does not take the message that seriously. This in turn, might lead to less strong feelings of discrete emotions like anger and worry than a humorous message containing a simile (Lazarus, 1991; Nabi et al., 2007).

Moreover, because recipients’ levels of excitative responses depend on the novelty and complexity of the humor, we could also expect differences in the arousal eliciting potential of humorous similes versus humorous hyperboles (Berlyne, 1972). If understanding humorous similes (vs. hyperboles) is more cognitively taxing because this form of humor is more complex and novel (Bowdle & Gentner, 2005; McQuarrie & Mick, 1996), humorous similes (vs. hyperboles) might induce higher levels of excitative responses in recipients. However, since we do not have steadfast empirical evidence as to how strongly or which emotional or excitative responses these different types of humorous satirical messages will elicit, we ask:

*RQ2*: To what extent does the consumption of different types of humorous messages (similes vs. hyperboles) cause different emotional and excitative responses?

Regarding recipients’ emotional responses, many studies show that the emotions evoked by a media stimulus can influence recipients’ attitudes (e.g., Lecheler et al., 2015). Feelings of anger for example, increases recipients’ support for policies designed to punish people responsible for the negative event (Kühne & Schemer, 2015). Worries about climate change or feelings of hope about tackling climate change can result in more support for climate change policies (Feldman & Hart, 2018) and feelings of mirth (i.e., happiness) are shown to create a willingness to engage with the situation at hand (Martin, 2014). Because we hypothesize that consuming humorous (vs. nonhumorous) messages will result in more positive feelings of happiness and hopefulness, we expect that consuming humorous messages will lead to more message-congruent attitudes. However, since we also hypothesize that consuming humorous (vs. nonhumorous) messages results in less negative feelings of anger and worry, we on the contrary, also expect that consuming humorous messages will lead to less message-congruent attitudes.

Concerning recipients’ excitative responses, research shows that the arousal levels evoked by a media stimulus can influence recipients’ attitudes (Renshon et al., 2015). The relationship between humor induced arousal and its persuasiveness is characterized by an inverted U-curve (Berlyne, 1972). When humor induces moderate levels of arousal, it is most persuasive, while it is least persuasive when humor induces too little or too much arousal (Berlyne, 1972). However, since it is unclear how strongly the consumption of humorous satirical messages elicits excitative responses, we do not yet now if the arousal levels evoked by consuming humorous (vs. nonhumorous) messages will positively or negatively influence recipients’ message congruent attitudes. Together, this results in the following hypothesis:

*H5: (a)* Anger, *(b)* worry, *(c)* happiness, *(d)* hopefulness, and *(e)* excitement function as mediators in the relationship between the consumption of humorous versus nonhumorous (satirical) messages and message-congruent attitudes.

### Methods

#### Design, participants, materials, procedure, measures, and data-analysis

To test *H3* to *H5* and *RQ2*, we again conducted three experiments using the same stimulus materials as in Experiments 1 to 3. Experiment 4 replicated the design of Experiment 1, and Experiments 5 and 6 replicated the design of Experiments 2 and 3. Sample sizes were determined based on the same power analysis as in Experiments 1 to 3. US participants were recruited at the same time, in the same way, and followed the same procedure as the participants in Experiments 1 to 3. See Table 1 for the demographic characteristics of each experiment.

Message-congruent attitudes (Experiment 4: *M* = 79.38, *SD *= 23.59; Experiment 5: *M* = 66.39, *SD *= 20.88; Experiment 6: *M* = 71.76, *SD *= 20.45) and perceived humor (Experiment 4: *M* = 3.25, *SD* = 1.96; Experiment 5: *M* = 2.91, *SD* = 2.04; Experiment 6: *M* = 3.14, SD = 2.01) were measured similarly to Experiments 1 to 3. Instead of asking items about recipients’ cognitive responses to the transcripts, we used various items to tap into recipients’ emotional and excitative responses to the transcripts. Discrete emotional (Nabi, 2010) and excitative responses to the transcripts were measured using single item measures asking participants on a seven-point Likert scale (1 = not at all to 7 = an extreme amount) to what extent they experienced feelings of anger (Experiment 4: *M* = 2.66, *SD* = 1.69; Experiment 5: *M* = 2.86, *SD* = 1.69; Experiment 6: *M* = 2.23, *SD* = 1.50), worry (Experiment 4: *M* = 3.71, *SD* = 1.75; Experiment 5: *M* = 3.25, *SD* = 1.78; Experiment 6: *M* = 3.01, *SD *= 1.70), happiness (Experiment 4: *M* = 1.54, *SD* = 1.04; Experiment 5: *M* = 1.57, *SD* = 1.11; Experiment 6: *M* = 1.47, *SD *= 1.00), hopefulness (Experiment 4: *M* = 1.78, *SD* = 1.22; Experiment 5: *M* = 1.84, *SD *= 1.30; Experiment 6: *M* = 1.50, *SD* = 1.03), and excitement (Experiment 4: *M* = 1.49, *SD* = 1.01; Experiment 5: *M* = 1.49, *SD* = 1.06; Experiment 6: *M* = 1.41, *SD* = 0.95) while reading the transcript.

The data analysis of Experiments 4 to 6 emulated Experiments 1 to 3, meaning we only report the results of the mini meta-analyses conducted to support the hypotheses in the article. The results of all individual analyses are reported in Appendix D, while the data and data analyses are reported in Appendix E.

### Results

#### Manipulation checks

Table 5 shows the differences in participants’ levels of perceived humor between all different (satirical) messages (statistical results of the analyses are presented in Appendix F). The results of our mini meta-analysis show that consuming humorous versus nonhumorous (satirical) messages resulted in higher levels of perceived humor (Table 3). Therefore, the manipulations were successful.

**Table 5.** Means and (SDs) of dependent variables across conditions of Experiments 4–6.

	Condition	*n*	Perceived Humor	Hopefulness	Happiness	Anger	Worry	Excitement	Attitudes
Experiment 4 Climate change (*N* = 489)	S	162	4.09 (1.88)^l^	1.93 (1.27)^l^	1.72 (1.18)^l^	2.31 (1.42)^l^	3.30 (1.58)^l^	1.60 (1.11)^l^	78.48 (23.76)^l^
	H	163	4.07 (1.75)^l^	1.69 (1.11)^l^	1.68 (1.09)^l^	2.50 (1.65)^l^	3.70 (1.79)^lm^	1.48 (1.04)^l^	80.32 (24.39)^l^
	NHS	164	1.62 (0.98)^m^	1.71 (1.27)^l^	1.22 (0.71)^m^	3.18 (1.85)^m^	4.13 (1.78)^m^	1.38 (0.86)^l^	79.35 (22.70)^l^
Experiment 5 Student loan debt (*N* = 543)	S	137	4.92 (1.74)^n^	1.81 (1.17)^n^	1.75 (1.17)^n^	2.57 (1.65)^n^	3.01 (1.73)^n^	1.58 (1.05)^n^	65.96 (22.35)^n^
	H	133	3.67 (1.92)°	1.87 (1.46)^n^	1.77 (1.20)^n^	2.72 (1.73)^n^	3.23 (1.84)^n^	1.56 (1.14)^n^	68.50 (20.77)^n^
	NHS	136	1.71 (1.03)^p^	1.91 (1.38)^n^	1.45 (1.15)^no^	3.31 (1.68)°	3.49 (1.85)^n^	1.45 (1.11)^n^	66.99 (19.49)^n^
	NHR	137	1.36 (0.68)^p^	1.76 (1.15)^n^	1.33 (0.83)°	2.85 (1.61)^no^	3.28 (1.70)^n^	1.36 (0.92)^n^	64.16 (20.78)^n^
Experiment 6 Brexit (*N* = 536)	S	134	4.30 (1.87)^q^	1.51 (0.99)^q^	1.66 (1.12)^q^	2.11 (1.37)^qr^	2.90 (1.66)^qr^	1.57 (1.11)^q^	69.04 (21.11)^q^
	H	133	4.16 (2.03)^q^	1.56 (1.13)^q^	1.71 (1.20)^q^	2.01 (1.40)^q^	2.68 (1.71)^q^	1.55 (1.09)^q^	72.12 (21.93)^q^
	NHS	134	2.34 (1.57)^r^	1.49 (1.02)^q^	1.26 (0.74)^r^	2.49 (1.64)^r^	3.29 (1.74)^r^	1.28 (0.75)^qr^	74.47 (18.47)^q^
	NHR	135	1.79 (1.16)^s^	1.44 (0.96)^q^	1.24 (0.76)^r^	2.30 (1.55)^qr^	3.15 (1.62)^qr^	1.24 (0.72)^r^	71.43 (20.02)^q^

S = humorous satirical message containing a simile; H = humorous satirical message containing an hyperbole; NHS = nonhumorous satirical message; NHR = nonhumorous regular news message. Expect for message-congruent attitudes, which was measured on a scale from 0 to 100, all variables were measured on 7-point scales. Different superscripts in the same column of each experiment indicate significant differences of at least *p* < .05. Each experiment uses its own set of superscripts.

#### Emotional and excitative responses

In relation to *H3*, the results of our mini meta-analysis show that consuming humorous (vs. nonhumorous) messages resulted in lower levels of anger and worry and in higher levels of happiness and excitement but not in higher levels of hopefulness (Table 3). Therefore, these results support *H3a, H3b*, and *H3c* but do not support *H3d*. In relation to *H4*, the results of our mini meta-analysis show that consuming humorous (vs. nonhumorous) messages resulted in higher levels of excitement see Table 3). Therefore, these results support *H4*.

Regarding *RQ3*, we found some small differences on participants’ emotional and excitative responses between the humorous similes and humorous hyperboles, but only in comparison to the nonhumorous satirical or regular news messages (see Table 5 and Table F1 in Appendix F). However, these differences are not consistent across the experiments, which means that overall, participants’ emotional and excitative responses between all the different types of (satirical) messages did not vary.

#### Message-congruent attitudes

The results of our mini meta-analysis show that consuming humorous (vs nonhumorous) messages did not directly result in more message-congruent attitudes (Table 3). We also found no significant differences in message-congruent attitudes between all the different types of (satirical) messages (see Table 5 and Table F1 in Appendix F).

**Figure 2.**
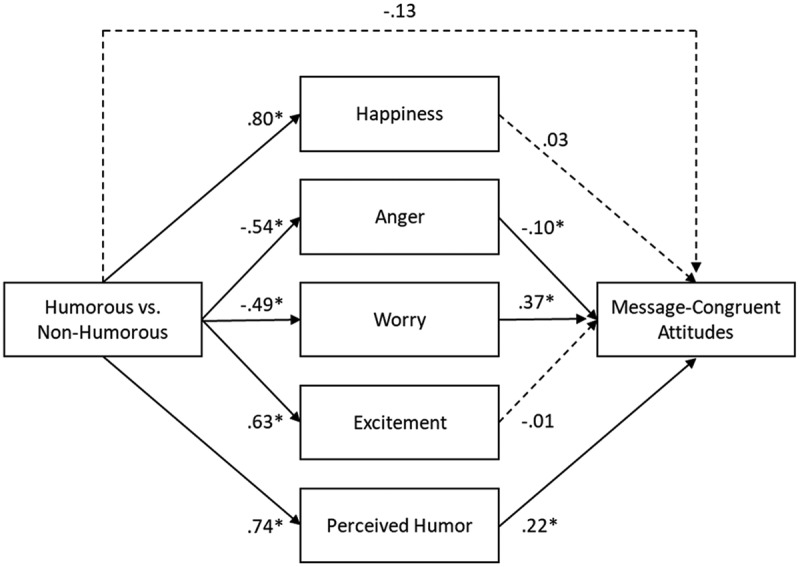
Meta-analysis of indirect effects Experiment 4–6 (emotional and excitative responses). Standardized regression estimates are shown. Paths with continuous lines are significant and paths with dotted lines are not significant. *p < .05, **p < .001.

Regarding *H5*, the results of our mini meta-analysis show that the relationship between consuming humorous versus nonhumorous messages and message-congruent attitudes was mediated through negative feelings of anger and worry and through perceived humor, but not through positive feelings of happiness and not through feelings of excitement. Therefore, results support *H5a* and *H5b* but do not support *H5c, H5d*, and *H5e*. Consuming humorous (vs. nonhumorous) messages again evoked opposing underlying processes that might have suppressed an overall effect on attitudes: On the one hand, consuming humorous (vs. nonhumorous) messages led to more perceived humor of the message and to less anger, which resulted in more message-congruent attitudes. On the other hand, consuming humorous (vs. nonhumorous) messages led to less worry, which resulted in less message agreement (Table 4 and Figure 2).

### Discussion

In line with our expectations, we found that recipients who read a humorous satirical message felt less angry (*H3a*) and worried (*H3b*) and felt happier (*H3c*) and more excited (*H4*) than recipients who read a nonhumorous (satirical) message. Thus, these findings show that resolving the humorous incongruity in a satirical message can lead to feelings of happiness and excitement (Berlyne, 1972; Eisend, 2009, 2011) and that the use of humor can soften the critical nature of a satirical message (Eisend, 2009, 2011).

However, contrary to our expectations (*H3d*), recipients who read a humorous satirical message did not feel more hopeful than recipients who read a nonhumorous (satirical) message (Peifer & Landreville, 2020). This finding can be explained by the fact that feelings of hope were low overall, indicating a possible floor effect. This means that the content of the messages might not have described the prospect of a desired and uncertain but possible future event (Droog et al., 2020), and the use of humor could thus probably not enhance these feelings of hope strong enough. This most likely resulted in too little variation in feelings of hope between the recipients in the humorous versus nonhumorous conditions.

Moreover, recipients’ attitudes were influenced through three underlying processes: perceived humor, anger, and worry. Consuming humorous versus nonhumorous messages led to more message-congruent attitudes because recipients perceived the messages as more humorous and because they felt less angry (*H5a*), while at the same time this consumption led to less message-congruent attitudes because recipients felt less worried (*H5b*). As a result, these responses may have partially suppressed an overall effect on attitudes. Moreover, although significant, some of these estimates were relatively weak, which suggests that the influence of these emotional responses on attitudes are subtle.

Contrary to our expectations, feelings of happiness (*H5c*) and excitement (*H5e*) did not mediate this relationship. This might be explained by the fact that both happiness and excitement scores were low overall. Feelings of happiness are shown to create a willingness to engage with the situation at hand (Martin, 2014), and humor is only persuasive when it induces moderate levels of arousal (Berlyne, 1972). Since the messages did not induce strong feelings of happiness and excitement, these feelings were probably not strong enough to impact recipients’ attitudes.

Moreover, stronger feelings of anger unexpectedly resulted in less message-congruent attitudes. This can be explained by the fact that anger motivates recipients to take immediate action to resolve the anger-evoking situation (Walter et al., 2018). Research shows that for persuasion to occur, it is critical for such messages to assist in achieving this emotion-induced goal (Nabi, 1999). This means that although our messages induced moderate levels of anger, the content of the messages might not have provided a “viable avenue to direct the activated anger into action” (Walter et al., 2018, p. 6), which resulted in an attitudinal backlash.

Moreover, our findings show no consistent differences in how different forms of humorous (similes vs. hyperboles) and nonhumorous (satirical news or regular news) messages influence recipients’ feelings of anger, worry, hopefulness, happiness, and excitement (*RQ2*). This means that the specific form through which the humorous criticism is delivered does not seem to influence which affective responses recipients feel at that moment (Bowdle & Gentner, 2005; McQuarrie & Mick, 1996; Rubio-Fernández et al., 2015).

## General discussion

Overall, the findings of our six experiments build on the theoretical framework of the DSMM by showing *how* the consumption of *different types* of satirical content can influence recipients’ attitudes, by demonstrating the indirect processes that underlie this relationship (Valkenburg & Peter, 2013). Our results show that the consumption of humorous versus nonhumorous (satirical) messages has a differential impact on all three mediator types distinguished by the DSMM (Valkenburg & Peter, 2013). However, only recipients’ levels of message discounting (cognitive response), feelings of anger and worry (emotional responses), and perceived humor (which contains both cognitive as well as emotional components) function as mediators in the relationship between satirical humor consumption and message-congruent attitudes. This means that recipients’ cognitive and emotional responses seem to play the most important role in affecting attitudes (Valkenburg & Peter, 2013).

Nevertheless, these cognitive and emotional responses elicited contrasting indirect effects. This finding can help explain some of the mixed persuasive effects found in the literature (Boukes et al., 2015), because these opposing co-occurring underlying mechanisms might suppresses the overall direct relationship between satirical news consumption and recipients’ attitudes. However, the causality between these cognitive, emotional and excitative responses and recipients’ attitudes should be further established by experimentally manipulating each of the underlying processes (e.g., inducing specific emotional states in recipients). This research can lead to a better understanding of the interplay between these cognitive, emotional, and excitative responses and recipients’ attitudes during (satirical) media consumption.

Moreover, the DSMM also argues that such mediating effects are conditional depending on various types of personal susceptibility variables (Valkenburg & Peter, 2013). This means that the way in which these underlying cognitive, emotional, and excitative responses to humorous versus nonhumorous (satirical) messages influence attitudes might vary across recipients. One of such susceptibility variables is recipients’ levels of need for cognition, which reflects their desire to engage in effortful cognitive activities (Cacioppo & Petty, 1982). From a resource allocation perspective, one could expect that recipients with high levels of need for cognition have more cognitive capacity left to counter-argue the humorous message. More counter-argumentation to the information in turn, might lead to less message agreement (Young, 2008). However, more research is needed to investigate how such recipient characteristics can act as moderators on the indirect persuasive effects of satirical news consumption.

Our findings also highlight the importance of distinguishing between the persuasive impact of inter- and intragenre levels of satirical news content. Our results demonstrate how the cognitive, emotional and excitative processing of different messages within the genre of satirical news (intragenre perspective) can differ from the processing of satirical news as a genre (intergenre perspective). This differential processing might also explain the mixed results found in the literature on an intergenre level. Research on satirical news as a genre for example did not provide consensus yet on how satirical news is cognitively and excitatively processed (Droog et al., 2023). Our intragenre level experiments provide more consistent results and demonstrate that recipients are more excited by and take the humorous information in a satirical news episode less seriously than the nonhumorous information in the same episode (Nabi et al., 2007). These contrasting mechanisms might prevent an overall effect on excitement and message discounting of satirical news consumption as a genre, because satirical news contains both humorous as well as nonhumorous information (Fox, 2018).

Moreover, research from an intergenre perspective consistently demonstrated that satire consumption can elicit negative emotions such as anger and worry (Chen et al., 2017; Droog et al., 2023). However, our intragenre level experiments showed that consuming humorous satirical messages actually diminish negative feelings of anger and worry compared to nonhumorous (satirical) messages. The differential emotional impact of the inter and intragenre levels of satirical news content can probably be explained by that fact that humans have an evolutionary tendency to attend to and be more influenced by negativity (i.e., the negativity bias; Kanouse & Hanson, 1972). Therefore, the use of humor in a satirical news episode might soften recipients’ negative emotional responses to a message on an intragenre level, but the negative emotional responses to the nonhumorous information in the same satirical news episode might dominate recipients’ emotional responses on an intergenre level.

These contrasting inter- and intragenre level findings have important implications for communication and satire theories, since they emphasize the need for theories such as the DSMM (Valkenburg & Peter, 2013) and the message discounting principle (Nabi et al., 2007) to distinguish between inter- and intragenre levels of media (i.e., satirical news) content. By including a specific content related factor that distinguishes between inter- and intragenre types of content, these theories should be able to more precisely explain and predict which specific types of (combinations of) media (i.e., satirical news) content lead to which specific types of responses in recipients.

In relation to even more specific intragenre related differences in satirical news content, our findings show that there are no consistent differences in the degree to which humorous satirical messages containing a simile versus hyperbole influence recipients’ cognitive, emotional and excitative responses. This is probably because these messages did not have a differential impact on recipients’ perceptions of the complexity and convincingness (i.e., sense of urgency) of these messages (see Appendix D). Therefore, the humorous similes probably did not require more cognitive effort to understand and recipients probably did not perceive them as more serious or as more of a threat to their personal goals than the humorous hyperboles (Bowdle & Gentner, 2005; Rubio-Fernández et al., 2015).

This might be explained by the narrative strategy of the humorous similes in our stimulus materials. The similes all contained an enriched narrative strategy in which the incongruity between the target and the source domain of the simile was resolved by a sentence explaining this relationship in detail (Droog et al., 2023). The grounds on which the comparison between the target and the source was based were therefore explicitly stated and not left to the audience for interpretation, which is why these humorous similes were probably not more complex to understand than the hyperbolical jokes. Future research should further unravel what factors attribute to differences in the perceived complexity and sense of urgency of different types of satirical messages, to provide more insight into the question when and why some types of satirical content may or may not be persuasive.

Some caveats should be noted about our experiments. First, in our manipulation, we showed our recipients a (fictional) transcript of satirical or regular news show instead of a short video clip in which the (satirical) host of the show could be seen and heard. Although this slightly lowers the external and ecological validity of our experiments, using (fictional) transcripts was necessary to establish internal validity of our experiments. This is because we needed to precisely construct all different (satirical) messages so that they all contained the same evaluative message. However, because we often relied on real American (satirical) news discourse to construct these different messages, the different messages still sometimes differed slightly in length. Moreover, we used societal issues as the subjects of our stimulus materials. Nevertheless, some scholars argue that when societal issues are discussed in American (satirical) news they are often linked to certain societal or political actors involved in that issue (Matthes & Rauchfleisch, 2013). We deliberately chose to direct our (satirical) messages only toward the societal issues of the stimulus materials without mentioning any societal or political actors, to make the stimulus materials less subject to change by news events surrounding those actors at the time of data collection. Therefore, we recommend replicating our experiments with different samples, different experimental materials, and in different modalities, to further test the robustness of our findings.

Overall, our results provide a better understanding of the underlying mechanisms that can explain when and why what type of satirical news consumption is persuasive or not. Although consuming humorous versus nonhumorous (satirical) messages differentially impacted almost all measured cognitive, emotional, and excitative responses, recipients’ attitudes were mainly influenced through their cognitive and emotional reactions. However, these mediating relationships often occurred in opposite directions, which might have suppressed an overall effect of the consumption of these humorous (vs nonhumorous) satirical messages on recipients’ attitudes. Moreover, our results also show the importance of distinguishing between satirical news content on an inter- and intragenre level. This distinction can help explain the mixed results found in the literature on an intergenre level, since the differential processing of both the humorous and nonhumorous messages in satirical news can elicit contrasting cognitive, emotional and excitative responses that can both enhance and impede the persuasiveness of satirical news, and thereby may prevent an overall persuasive effect of satirical news consumption.

## Supplementary Material

Supplemental Material

Supplemental Material

Supplemental Material

Supplemental Material

Supplemental Material

## Data Availability

The data that support the findings of this study are openly available on the Open Science Framework https://edu.nl/a6hh3.

## Notes

1. The study and analysis plan of these experiments were not preregistered.

2. We also measured participants’ level of perceived harshness, complexity, convincingness, novelty (Experiments 2, 3, 5, and 6) and aptness (Experiments 2, 3, 5, and 6) of the transcripts, but the results of these variables are not reported in this article.

3. Before conducting the main analyses of all the experiments, we first conducted control analyses to investigate if we needed to include certain demographic or control variables in our analyses. The results of these control analyses are presented in Appendix B. We also conducted correlation analyses to investigate the general relationships between all the dependent and control variables. These correlation tables are presented in Appendix C.

4. We included resource allocation in this meta-analytical mediation analysis because in Experiment 2 resource allocation was significantly impacted by the consumption of humorous versus nonhumorous messages.
