# Supplementary material for: The underlying mechanisms of the persuasiveness of different types of satirical news messages
Source: Discourse Process. 2024 Aug 20;61(10):479–97. doi: 10.1080/0163853X.2024.2381407 (PMC11614037; doi:10.1080/0163853X.2024.2381407)
Supplement: Supplemental Material [file HDSP_A_2381407_SM9830.pdf]

Appendix A: Stimulus Materials

Table A1.

Transcripts of stimulus materials used in all experiments.

Issue	Humorous satirical message using a simile	Humorous satirical message using a hyperbole	Non-humorous satirical message	Non-humorous regular news message
Climate change<br><br>Exp. 1&4	>> Trevor Noah: Let's start our show with climate change. Humans are increasingly influencing the climate and the Earth's temperature by burning fossil fuels, cutting down forests and farming livestock. Climate change, it is the gradual disaster that will eventually wipe us out if we don't take it seriously. **Think of it like oatmeal. You know, if you wash your bowl right away: no problem! But... If you leave it in the sink, by the time you come home, you will have a new roommate.**	>> Trevor Noah: Let's start our show with climate change. Humans are increasingly influencing the climate and the Earth's temperature by burning fossil fuels, cutting down forests and farming livestock. Climate change, it is the gradual disaster that will eventually wipe us out if we don't take it seriously. **Experts predict that if we do nothing about it, it will become so hot that chickens are going to lay hard-boiled eggs.**	>> Trevor Noah: Let's start our show with climate change. Humans are increasingly influencing the climate and the Earth's temperature by burning fossil fuels, cutting down forests and farming livestock. Climate change, it is the gradual disaster that will eventually wipe us out if we don't take it seriously. **Experts predict that large parts of the planet will become inhabitable if we do nothing about it.**	
Student loan debt<br><br>Exp. 2&5	>> Trevor Noah: Tonight, we are talking about student loan debt. Student loans are affecting millions of people, many of them just out of college. Therefore, the government should take action to resolve this long-time national issue. One of the things that makes student loan debt such a problem for the economy is that it's so difficult to repay your student debt in full. **Student loan debt is like the new herpes. Almost everybody has it, it stays with you your whole life, and eventually you're gonna have to tell your partner about it.**	>> Trevor Noah: Tonight, we are talking about student loan debt. Student loans are affecting millions of people, many of them just out of college. Therefore, the government should take action to resolve this long-time national issue. One of the things that makes student loan debt such a problem for the economy is that it's so difficult to repay your student debt in full. **Most student loans take so long to repay, that many of you will still be paying off your loans after you have been in heaven for a thousand years.**	>> Trevor Noah: Tonight, we are talking about student loan debt. Student loans are affecting millions of people, many of them just out of college. Therefore, the government should take action to resolve this long-time national issue. One of the things that makes student loan debt such a problem for the economy is that it's so difficult to repay your student debt in full. **Most student loans take so long to repay that many of you will still be paying of your loans well into middle age.**	>> **Lester Holt** : Tonight, we are talking about student loan debt. Student loans are affecting millions of people, many of them just out of college. Therefore, the government should take action to resolve this long-time national issue. One of the things that makes student loan debt such a problem for the economy is that it's so difficult to repay your student debt in full. **Most student loans take so long to repay that many of you will still be paying of your loans well into middle age.**
Brexit<br><br>Exp. 3&6	>> Trevor Noah: Our main story tonight concerns an issue that has often been in the news for the last years: Brexit. Brexit is the fun name that's been given to a disaster. Brexit has been an economic catastrophe for Britain, leading to empty supermarket shelves, a shortage of truck drivers, a fuel supply crisis, and nursing shortages in the country's hospitals. **The consequences of Brexit are a lot like Shrek. Quite messy, really complicated and very bad-tempered.**	>> Trevor Noah: Our main story tonight concerns an issue that has often been in the news for the last years: Brexit. Brexit is the fun name that's been given to a disaster. Brexit has been an economic catastrophe for Britain, leading to empty supermarket shelves, a shortage of truck drivers, a fuel supply crisis, and nursing shortages in the country's hospitals. **The consequences of Brexit are so bad, that British bookstores have relabeled their post-apocalyptical fiction section as "Current British Affairs".**	>> Trevor Noah: Our main story tonight concerns an issue that has often been in the news for the last years: Brexit. Brexit is the fun name that's been given to a disaster. Brexit has been an economic catastrophe for Britain, leading to empty supermarket shelves, a shortage of truck drivers, a fuel supply crisis, and nursing shortages in the country's hospitals. **The consequences of Brexit are so bad that they are expected to negatively impact Britain long into the future.**	>> **Lester Holt** : Our main story tonight concerns an issue that has often been in the news for the last years: Brexit. Brexit is the fun name that's been given to a disaster. Brexit has been an economic catastrophe for Britain, leading to empty supermarket shelves, a shortage of truck drivers, a fuel supply crisis, and nursing shortages in the country's hospitals. **The consequences of Brexit are so bad that they are expected to negatively impact Britain long into the future.**

Figure A1.

Examples of stimulus materials as shown to the participants.

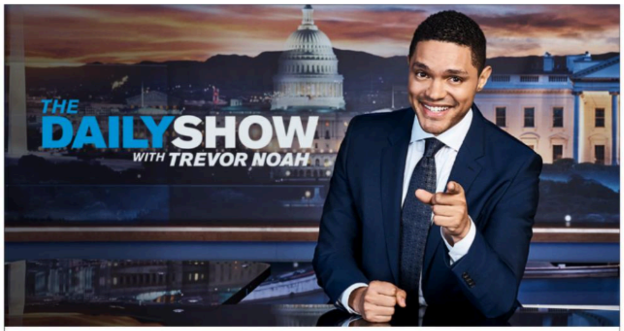

[00:01]

>> Trevor Noah: Let’s start our show with climate change. Humans are increasingly influencing the climate and the Earth's temperature by burning fossil fuels, cutting down forests and farming livestock.

Climate change, it is the gradual disaster that will eventually wipe us out if we don’t take it seriously. Think of it like oatmeal. You know, if you wash your bowl right away: no problem! But… If you leave it in the sink, by the time you come home, you will have a new roommate.

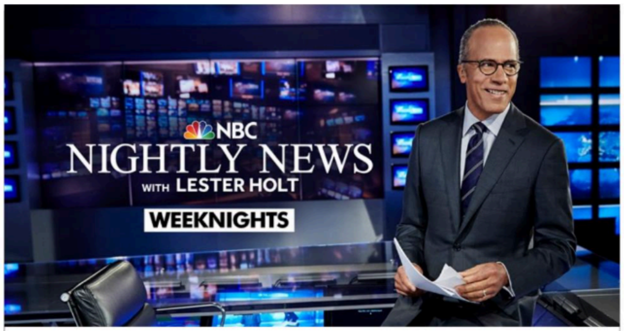

[00:01]

>> Lester Holt: Our main story tonight concerns an issue that has often been in the news for the last years: Brexit. Brexit is the fun name that's been given to a disaster. Brexit has been an economic catastrophe for Britain, leading to empty supermarket shelves, a shortage of truck drivers, a fuel supply crisis, and nursing shortages in the country’s hospitals.

The consequences of Brexit are so bad that they are expected to negatively affect Britain long into the future.
