# Supplementary material for: The underlying mechanisms of the persuasiveness of different types of satirical news messages
Source: Discourse Process. 2024 Aug 20;61(10):479–97. doi: 10.1080/0163853X.2024.2381407 (PMC11614037; doi:10.1080/0163853X.2024.2381407)
Supplement: Supplemental Material [file HDSP_A_2381407_SM9829.pdf]

Appendix B: Control Analyses

We first established whether the experimental conditions across the different experiments did not differ on any of the demographic and control variables.

Experiment 1 (climate change)

Participants were equally distributed across the three experimental conditions (humorous simile vs. humorous hyperbole vs. non-humorous satirical message) in regard to age ($F(2, 483) = 0.84$, $p = .432$), gender ($\chi^2(2) = 0.47$, $p = .790$), educational level ($\chi^2(16) = 11.98$, $p = .746$), political ideology ($F(2, 483) = 1.99$, $p = .138$), source liking ($F(2, 483) = 1.19$, $p = .304$), satirical news consumption ($F(2, 483) = 3.00$, $p = .051$) and regular news consumption ($F(2, 483) = 1.32$, $p = .268$). Because the randomization was successful we did not include any of these demographics and control variables as covariates in the analyses.

Experiment 2 (student loan debt)

Participants were equally distributed across the four experimental conditions (humorous simile vs. humorous hyperbole vs. non-humorous satirical message vs. non-humorous regular news message) in regard to age ($F(3, 540) = 0.24$, $p = .866$), gender ($\chi^2(3) = 0.69$, $p = .877$), educational level ($\chi^2(24) = 22.85$, $p = .529$), political ideology ($F(3, 540) = 0.17$, $p = .918$), source liking ($F(3, 540) = 0.55$, $p = .649$), satirical news consumption ($F(3, 540) = 1.21$, $p = .306$) and regular news consumption ($F(3, 540) = 1.19$, $p = .313$). Because the randomization was successful we did not include any of these demographics and control variables as covariates in the analyses.

Experiment 3 (Brexit)

Participants were equally distributed across the four experimental conditions (humorous simile vs. humorous hyperbole vs. non-humorous satirical message vs. non-humorous regular news message) in regard to age ($F(3, 537) = 0.93$, $p = .428$), gender ($\chi^2(3) = 1.08$, $p = .783$), educational level ($\chi^2(24) = 32.73$, $p = .110$), political ideology ($F(3, 537) = 1.59$, $p = .181$), source liking ($F(3, 537) = 0.48$, $p = .696$), and regular news consumption ($F(3, 537) = 1.61$, $p = .187$). However, participants were not equally distributed among the experimental conditions in terms of satirical news consumption ($F(3, 537) = 3.72$, $p = .011$). Therefore we ran the analyses with and without satirical news consumption as a covariate. Using satirical news consumption as a covariate did not yield different results. Therefore the analyses in the main manuscript are reported without satirical news consumption as a covariate.

Experiment 4 (climate change)

Participants were equally distributed across the three experimental conditions (humorous simile vs. humorous hyperbole vs. non-humorous satirical message) in regard to age ($F(2, 486) = 0.49, p = .615$), gender ($\chi^2(2) = 3.18, p = .204$), educational level ($\chi^2(14) = 17.38, p = .236$), political ideology ($F(2, 486) = 0.24, p = .786$), source liking ($F(2, 486) = 2.80, p = .062$), satirical news consumption ($F(2, 486) = 1.26, p = .284$) and regular news consumption ($F(2, 486) = 1.11, p = .330$). Because the randomization was successful we did not include any of these demographics and control variables as covariates in the analyses.

Experiment 5 (student loan debt)

Participants were equally distributed across the four experimental conditions (humorous simile vs. humorous hyperbole vs. non-humorous satirical message vs. non-humorous regular news message) in regard to age ($F(3, 539) = 0.51, p = .677$), gender ($\chi^2(3) = 2.46, p = .482$), educational level ($\chi^2(24) = 25.94, p = .356$), political ideology ($F(3, 539) = 1.10, p = .351$), source liking ($F(3, 539) = 0.55, p = .649$), and satirical news consumption ($F(3, 539) = 0.37, p = .778$). However, participants were not equally distributed among the experimental conditions in terms of regular news consumption ($F(3, 539) = 3.03, p = .029$). Therefore we ran the analyses with and without regular news consumption as a covariate. Using regular news consumption as a covariate did not yield different results. Therefore the analyses in the main manuscript are reported without regular news consumption as a covariate.

Experiment 6 (Brexit)

Participants were equally distributed across the four experimental conditions (humorous simile vs. humorous hyperbole vs. non-humorous satirical message vs. non-humorous regular news message) in regard to age ($F(3, 532) = 0.74, p = .527$), educational level ($\chi^2(24) = 28.90, p = .224$), political ideology ($F(3, 532) = 0.16, p = .924$), source liking ($F(3, 532) = 1.08, p = .356$), satirical news consumption ($F(3, 532) = 0.75, p = .522$) and regular news consumption ($F(3, 532) = 1.53, p = .205$). However, participants were not equally distributed among the experimental conditions in terms of gender ($\chi^2(3) = 8.84, p = .032$). Therefore we ran the analyses with and without gender as a covariate. Using gender as a covariate did not yield different results. Therefore the analyses in the main manuscript are reported without gender consumption as a covariate.
