# Supplementary material for: The underlying mechanisms of the persuasiveness of different types of satirical news messages
Source: Discourse Process. 2024 Aug 20;61(10):479–97. doi: 10.1080/0163853X.2024.2381407 (PMC11614037; doi:10.1080/0163853X.2024.2381407)
Supplement: Supplemental Material [file HDSP_A_2381407_SM9828.pdf]

Appendix C: Correlation Tables Dependent Variables

Table C1

Correlations Between Dependent Variables and control variables Experiment 1.

	1.	2.	3.	4.	5.	6.	7.	8.	9.	10.	11.
1. Perceived Humor	-										
2. Message Discounting	.37**	-									
3. Resource Allocation	.00	.33**	-								
4. Message-Congruent Attitudes	.22**	-.17**	-.10*	-							
5. Age	-.05	-.05	.05	-.17**	-						
6. Gender	-.08	-.01	-.01	-.06	-.02	-					
7. Educational Level	.04	-.09*	-.01	.06	.21**	.05	-				
8. Political Ideology	-.23**	.05	.18**	-.58**	.22**	.06	-.09	-			
9. Source Liking	.32**	-.17**	-.15**	.50**	-.01	-.14**	.06	-.40**	-		
10. Regular News Consumption	-.04	-.04	.04	.02	.15**	.00	.01	.02	.08	-	
11. Satirical News Consumption	.21**	-.05	-.08	.31**	.01	-.02	.12**	-.32**	.43**	.47**	-

Note. * p < .05. ** p < .01.

Table C2

Correlations Between Dependent Variables and control variables Experiment 2.

	1.	2.	3.	4.	5.	6.	7.	8.	9.	10.	11.
1. Perceived Humor	-										
2. Message Discounting	.53**	-									
3. Resource Allocation	-.10*	.17**	-								
4. Message-Congruent Attitudes	.10*	-.14**	-.06	-							
5. Age	-.02	-.07	.06	-.23**	-						
6. Gender	-.01	-.02	-.09*	-.11*	.03	-					
7. Educational Level	.05	.03	.04	-.05	.10*	.04	-				
8. Political Ideology	-.07	.15**	.12**	-.30**	.17**	-.03	-.12**	-			
9. Source Liking	.24**	-.14**	-.13**	.38**	.04	-.11*	.09*	.37**	-		
10. Regular News Consumption	.07	-.02	.03	.03	.39**	-.07	.03	.09*	.18**	-	
11. Satirical News Consumption	.20**	-.03	-.11**	.16**	.05	.05	.11**	-.27**	.42**	.43**	-

Note. * $p < .05$. ** $p < .01$.

Table C3

Correlations Between Dependent Variables and control variables Experiment 3.

	1.	2.	3.	4.	5.	6.	7.	8.	9.	10.	11.
1. Perceived Humor	-										
2. Message Discounting	.52**	-									
3. Resource Allocation	.01	.28**	-								
4. Message-Congruent Attitudes	.14**	-.08	-.06	-							
5. Age	.03	-.03	-.01	-.13**	-						
6. Gender	-.02	-.06	-.14*	-.14**	.01	-					
7. Educational Level	.06	.01	-.03	-.02	.15**	.04	-				
8. Political Ideology	-.26**	-.05	.14**	-.36**	.24**	.01	-.08	-			
9. Source Liking	.35**	.06	-.13**	.51**	-.01	-.10**	.07	-.42**	-		
10. Regular News Consumption	.12**	.08	.01	.01	.29**	-.05	.06	.11*	.14**	-	
11. Satirical News Consumption	.30**	.11*	-.2**	.24**	.00	.07	.10*	-.27**	.44**	.47**	-

Note. * $p < .05$. ** $p < .01$.

Table C4

Correlations Between Dependent Variables and control variables Experiment 4.

	1.	2.	3.	4.	5.	6.	7.	8.	9.	10.	11.	12.	13.	14.
1. Perceived Humor	-													
2. Hopefulness	.06	-												
3. Happiness	.31**	.44**	-											
4. Anger	-.16**	.13**	-.10*	-										
5. Worry	.01	.17**	-.03	.55**	-									
6. Excitement	.18**	.34**	.61**	.08	.11*	-								
7. Message-Congruent Attitudes	.16**	.02	.05	.15**	.50**	.10*	-							
8. Age	-.09	.09	.02	-.02	-.10**	.04	-.19**	-						
9. Gender	.03	.09	.03	-.07	-.15*	.08	-.13**	.01	-					
10. Educational Level	.12**	-.07	.06	-.02	.02	.03	.01	.13**	-.02	-				
11. Political Ideology	-.19**	.06	.01	-.14**	-.37**	-.04	-.61**	.22**	.03	-.09*	-			
12. Source Liking	.23**	.11*	.23**	.07	.36**	.16**	.51**	-.02	-.15**	.13**	-.46**	-		
13. Regular News Consumption	.01	.16**	.14**	-.01	.05	.13**	.01	.26**	-.09	.06	.12*	.05	-	
14. Satirical News Consumption	.28**	.09*	.17**	.05	.15**	.18**	.23**	.02	.01	.17**	-.21**	.34**	.44**	-

Note. * p < .05. ** p < .01.

Table C5

Correlations Between Dependent Variables and control variables Experiment 5.

	1.	2.	3.	4.	5.	6.	7.	8.	9.	10.	11.	12.	13.	14.
1. Perceived Humor	-													
2. Hopefulness	.04	-												
3. Happiness	.27**	.51**	-											
4. Anger	-.13**	-.05	-.10*	-										
5. Worry	-.08	.05	-.04	.62**	-									
6. Excitement	.16**	.59**	.75**	-.01	.02	-								
7. Message-Congruent Attitudes	.10*	.11*	.11*	.20**	.39**	.09*	-							
8. Age	-.06	-.10*	-.13**	-.10*	-.21**	-.10*	-.26**	-						
9. Gender	-.09*	-.05	.00	.06	-.08	.00	-.16**	.08	-					
10. Educational Level	.04	-.04	.01	.00	.01	.01	-.05	.22**	-.07	-				
11. Political Ideology	-.11**	.10	-.02	-.12**	-.21**	-.05	-.39**	.08	.08	-.14**	-			
12. Source Liking	.25**	.18**	.18**	.04	.19**	.13**	.35**	.00	-.19**	.07	-.30**	-		
13. Regular News Consumption	.00	.11*	.10*	.04	.05	.13**	.00	.19**	-.02	.04	.09*	.11*	-	
14. Satirical News Consumption	.15**	.10*	.13**	.11**	.12**	.13**	.22**	.10*	.02	.12**	-.28**	.35**	.48**	-

Note. * p < .05. ** p < .01.

Table C6

Correlations Between Dependent Variables and control variables Experiment 6.

	1.	2.	3.	4.	5.	6.	7.	8.	9.	10.	11.	12.	13.	14.
1. Perceived Humor	-													
2. Hopefulness	.03	-												
3. Happiness	.34**	.48**	-											
4. Anger	-.11*	.19**	-.01	-										
5. Worry	-.16**	.17**	-.05	.55**	-									
6. Excitement	.33**	.37**	.60**	14**	.08	-								
7. Message-Congruent Attitudes	.11*	.00	.03	.07	.20**	.03	-							
8. Age	-.11*	-.02	-.03	-.07	-.18**	-.08	-.10*	-						
9. Gender	-.02	-.02	.02	-.03	-.14**	-.06	-.06	-.01	-					
10. Educational Level	.04	-.07	.02	.01	.00	.03	-.07	.14**	-.01	-				
11. Political Ideology	-.20**	.06	-.04	.00	-.06	-.02	-.36**	.19**	-.01	-.07	-			
12. Source Liking	.25**	.12**	.19**	-.03	.09*	.13**	.44**	.05	-.12**	.08	-.46**	-		
13. Regular News Consumption	-.04	.23*	.07	.08	.13**	.10*	.06	.26**	-.10*	-.04	.12**	.13**	-	
14. Satirical News Consumption	.22**	.20**	.27**	.05	.13**	.23**	.20**	.03	-.08	.08	-.31**	.42**	.39**	-

Note. * p < .05. ** p < .01.
