# Supplementary material for: The underlying mechanisms of the persuasiveness of different types of satirical news messages
Source: Discourse Process. 2024 Aug 20;61(10):479–97. doi: 10.1080/0163853X.2024.2381407 (PMC11614037; doi:10.1080/0163853X.2024.2381407)
Supplement: Supplemental Material [file HDSP_A_2381407_SM9827.pdf]

Appendix D: Results of Analyses per Experiment

Manipulation check

Across all six experiments we consistently found that participants who read a humorous satirical message reported higher levels of perceived humor than participants who read a non-humorous (satirical) message (see Table D1 and D2 for details).

Cognitive Responses

We found that participants who read a humorous satirical message about climate change in Experiment 1, student loan debt in Experiment 2 or Brexit in Experiment 3, reported higher levels of message discounting than participants who read a non-humorous (satirical) message about these issues. Participants who read a humorous satirical message about climate change in Experiment 1 or Brexit in Experiment 3, did not report higher levels of resource allocation than participants who read a non-humorous (satirical) message about these issues, only participants who read humorous satirical message about student loan debt in Experiment 2, reported lower levels of resource allocation than participants who read a non-humorous (satirical) message about this issue (see Table D1 and D2).

Emotional Responses

We found that participants who read a humorous satirical message about climate change in Experiment 4, student loan debt in Experiment 5 and Brexit in Experiment 6, did not report higher levels of hopefulness than participants who read a non-humorous (satirical) message about these issues, while they did report higher levels of happiness and lower levels of anger than participants who read a non-humorous (satirical) message about these issues. Moreover, participants who read a humorous satirical message about climate change in Experiment 4 or Brexit in Experiment 6, reported lower levels of worry than participants who read a non-humorous (satirical) message, while there was no significant difference in levels of worry between these two conditions for messages about student loan debt in Experiment 5 (see Table D1 and D2 for details).

Excitative Responses

We found that participants who read a humorous satirical message about climate change in Experiment 4 or student loan debt in Experiment 5, did not report higher levels of excitement than participants who read a non-humorous (satirical) message about these issues, while participants who read humorous satirical message about Brexit in Experiment

6, did report higher levels of excitement than participants who read a non-humorous (satirical) message about this issue (see Table D1 and D2 for details).

Message-Congruent Attitudes

Direct Effects

In relation to participants' message-congruent attitudes, we found that in five of the six experiments, participants who read a humorous satirical message did not report higher levels of message-congruent attitudes than participants who read a non-humorous (satirical) message about these issues. Only participants who read a humorous satirical message about climate change in Experiment 4 reported higher levels of message-congruent attitudes than participants who read a non-humorous (satirical message) about this issue (see Table D1 and D2 for details).

Indirect Effects

Our mediation analyses consistently showed significant indirect effects of the consumption of humorous vs. non-humorous (satirical) messages about climate change in Experiment 1, student loan debt in Experiment 2 and Brexit in Experiment 3, on message-congruent attitudes, through message discounting and perceived humor of the messages but not though resource allocation (see Table D3). However, the consumption of the humorous (vs. non-humorous) messages evoked two opposing underlying processes that suppressed an overall direct effect on attitudes. The consumption of the humorous (vs. non-humorous) messages led to more agreement with the (satirical) messages, because participants perceived these messages as more humorous, while at the same time, the consumption of the humorous (vs. non-humorous) messages led to less agreement with the (satirical) messages, because participants discounted these messages more.

However, the analyses showed some more inconsistent indirect effects through happiness, anger, worry, excitement and perceived humor (see Table D4). The relationship between humorous (vs. non-humorous) messages about climate change in Experiment 4 on message-congruent attitudes is mediated through feelings of anger, worry and perceived humor, but not through feelings of happiness. Again, the consumption of the humorous (vs. non-humorous) messages evoked opposing underlying processes that suppressed an overall direct effect on attitudes. The consumption of the humorous (vs. non-humorous) messages about climate change in Experiment 4, led to more agreement with the (satirical) messages, because participants were less angry and perceived these messages as more humorous,

while at the same time, the consumption of the humorous (vs. non-humorous) messages led to less agreement with the (satirical) messages, because participants were less worried.

In addition, the relationship between humorous (vs. non-humorous) messages about student loan debt in Experiment 5 on message-congruent attitudes is mediated through feelings of happiness and anger, and not through feelings of worry and perceived humor. These opposing mediating effects occurred as follows: the consumption of the humorous (vs. non-humorous) messages about student loan debt in Experiment 5, led to more agreement with the (satirical) messages, because participants were happier, while at the same time, the consumption of the humorous (vs. non-humorous) messages led to less agreement with the (satirical) messages, because participants were less angry.

Moreover, the relationship between humorous (vs. non-humorous) messages about Brexit in Experiment 6 on message-congruent attitudes is mediated through feelings of worry and perceived humor, but not through feelings of happiness, anger and excitement. These opposing mediating effects occurred in the same way as with the messages about student loan debt in Experiment 5. The consumption of the humorous (vs. non-humorous) messages about Brexit in Experiment 6 led to more agreement with the (satirical) messages, because participants perceived these messages as more humorous, while at the same time, the consumption of the humorous (vs. non-humorous) messages about Brexit in Experiment 6, led to less agreement with the (satirical) messages, because participants were less worried.

Complexity and Convincingness

To rule out alternative explanations for the differences in the consumption of humorous satirical messages containing a simile versus hyperbole and the differences in the consumption of non-humorous satirical versus regular news messages on recipients' cognitive, emotional and excitative responses, we also investigated how complex and convincing participants perceived these messages to be (on 7-point Likert scales from 1 = completely disagree, to 7 = completely agree). Here we found that there were no significant differences in complexity scores between these different types of (satirical) messages across all six experiments (Exp. 1: $F(2, 483) = 0.95$, $p = .388$; Exp. 2: $F(3, 540) = 0.72$, $p = .540$; Exp. 3: $F(3, 537) = 1.44$, $p = .229$; Exp. 4: $F(2, 486) = 2.12$, $p = .121$; Exp. 5: $F(3, 539) = 0.99$, $p = .396$; Exp. 6: $F(3, 532) = 0.03$, $p = .994$). Moreover, in five of the six experiments there were also no significant differences in the convincingness scores between these different types of

(satirical) messages (Exp. 1: $F(2, 483) = 0.99$, $p = .373$; Exp. 2: $F(3, 540) = 2.21$, $p = .09$; Exp. 3: $F(3, 537) = 2.27$, $p = .080$; Exp. 4: $F(2, 486) = 3.01$, $p = .050$; Exp. 5: $F(3, 539) = 1.44$, $p = .231$). Only in Experiment 6, we found a significant difference between the different types of (satirical) messages (Exp. 6: $F(3, 532) = 3.95$, $p = 0.008$). In this experiment only the non-humorous satirical message was perceived as more convincing than the humorous simile. So overall this means that all the different types of (satirical) messages do not consistently differ on their complexity and convincingness.

Table D1.

Means and (Standard Deviations) of dependent variables across humorous and non-humorous conditions in each experiment.

	Climate Change (N = 486)		Student Loan Debt (N = 544)		Brexit (N = 541)	
Experiment 1-3	Humorous (*n* = 323)	Non-Humorous (*n* = 163)	Humorous (*n* = 271)	Non-Humorous (*n* = 273)	Humorous (*n* = 270)	Non-Humorous (*n* = 271)
Message Perceptions						
Perceived Humor	4.67 (1.58)^{a}	2.25 (1.42)^{b}	4.92 (1.59)^{a}	1.89 (1.15)^{b}	4.73 (1.47)^{a}	2.62 (1.51)^{b}
Cognitive Responses						
Message Discounting	3.14 (1.22)^{a}	2.19 (1.05)^{b}	3.13 (1.18)^{a}	2.01 (0.84)^{b}	3.66 (1.18)^{a}	2.52 (1.06)^{b}
Resource Allocation	2.22 (1.05)^{a}	2.19 (1.17)^{a}	1.96 (0.84)^{a}	2.24 (1.14)^{b}	2.32 (1.08)^{a}	2.28 (1.03)^{a}
Persuasion						
Message-Congruent Attitudes	82.70 (19.70)^{a}	78.23 (25.31)^{b}	66.73 (20.20)^{a}	65.07 (20.85)^{a}	69.00 (20.80)^{a}	70.46 (20.98)^{a}
	Climate Change (N = 489)		Student Loan Debt (N = 543)		Brexit (N = 536)	
Experiment 4-6	Humorous (*n* = 325)	Non-Humorous (*n* = 164)	Humorous (*n* = 270)	Non-Humorous (*n* = 273)	Humorous (*n* = 267)	Non-Humorous (*n* = 269)
Message Perceptions						
Perceived Humor	4.08 (1.81)^{a}	1.62 (0.98)^{b}	4.30 (1.93)^{a}	1.53 (0.89)^{b}	4.23 (1.95)^{a}	2.06 (1.40)^{b}
Emotional Responses						
Hopefulness	1.81 (1.19)^{a}	1.71 (1.27)^{a}	1.84 (1.32)^{a}	1.84 (1.27)^{a}	1.54 (1.06)^{a}	1.46 (0.99)^{a}
Happiness	1.70 (1.14)^{a}	1.22 (0.71)^{b}	1.76 (1.18)^{a}	1.39 (1.00)^{b}	1.69 (1.16)^{a}	1.25 (0.74)^{b}
Anger	2.41 (1.54)^{a}	3.18 (1.85)^{b}	2.64 (1.69)^{a}	3.08 (1.66)^{b}	2.06 (1.38)^{a}	3.39 (1.59)^{b}
Worry	3.50 (1.70)^{a}	4.13 (1.78)^{b}	3.12 (1.78)^{a}	3.38 (1.78)^{a}	2.79 (1.69)^{a}	3.22 (1.68)^{b}
Excitative Responses						
Excitement	1.54 (1.08)^{a}	1.38 (0.86)^{a}	1.57 (1.09)^{a}	1.40 (1.02)^{a}	1.56 (1.10)^{a}	1.56 (1.10)^{b}
Persuasion						
Message-Congruent Attitudes	79.40 (24.06)^{a}	79.35 (22.70)^{a}	67.21 (21.58)^{a}	65.57 (20.16)^{a}	70.58 (21.54)^{a}	70.58 (21.54)^{a}

Note. Except for message-congruent attitudes, which was measured on a scale from 0 to 100, all variables were measured on 7-point scales. Different superscripts in the same row indicate significant differences of at least $p < .05$.

Table D2.

Results of Independent T-Tests between humorous vs. non-humorous (satirical) messages on dependent variables in each experiment.

Experiment 1-3		Climate Change				Student Loan Debt				Brexit			
IV	DV	*df*	*t*	*p*	*d*	*df*	*t*	*p*	*d*	*df*	*t*	*p*	*d*
Humorous vs. Non-Humorous (satirical) messages	Message Perceptions												
	Perceived Humor	484	16.43	< .001**	1.58	490.52	25.37	< .001**	2.18	482.92	14.77	< .001**	1.34
	Cognitive Responses												
	Message Discounting	370.88	8.90	< .001**	0.81	542	12.68	< .001**	1.09	539	11.78	< .001**	1.01
	Resource Allocation	484	0.21	.834	0.02	542	-3.29	< .05*	-0.28	539	0.64	.636	0.04
	Persuasion												
	Message-Congruent Attitudes	263.81	1.98	< .05*	0.21	542	0.94	.346	0.08	539	-0.81	.417	-0.07
**Experiment 4-6**		Climate Change				Student Loan Debt				Brexit			
IV	DV	*df*	*t*	*p*	*d*	*df*	*t*	*p*	*d*	*df*	*t*	*p*	*d*
Humorous vs. Non-Humorous (satirical) messages	Message Perceptions												
	Perceived Humor	485.12	19.51	< .001**	1.56	377.47	21.42	< .001**	1.85	534	11.78	< .001**	1.28
	Emotional Responses												
	Hopefulness	487	0.82	.413	0.08	541	0.05	.960	0.00	534	0.80	.425	0.07
	Happiness	465.58	5.71	< .001**	0.47	525.02	3.95	< .001**	0.34	452.77	5.18	< .001**	0.45
	Anger	278.73	-4.59	< .001**	-0.47	541	-3.03	< .05*	-0.26	524.74	-2.58	< .05*	-0.22
	Worry	487	-3.80	< .001**	-0.36	541	-1.69	.091	-0.15	534	-2.92	<. 05*	-0.25
	Excitative Responses												
	Excitement	396.95	1.75	.104	0.16	537.81	1.85	.065	0.16	462.31	3.78	< .001**	0.33
	Persuasion												
	Message-Congruent Attitudes	487	0.20	.984	0.00	541	0.92	.360	0.08	534	-1.34	.181	-0.12

Note. * Significant at the .05 level. ** Significant at the 0.001 level.

Table D3.

Mediation analysis on the relationship between humorous satirical vs. non-humorous (satirical) messages on message-congruent attitudes through cognitive responses.

Climate Change								
				95% C.I.				
Type	Effect	Estimate	*SE*	Lower	Upper	*t*	*p*	β
Component	C → MD	0.95	0.11	0.73	1.17	8.48	< .001**	0.76
	C → PH	2.41	0.15	2.13	2.70	16.43	< .001**	1.27
	MD → MCA	-5.09	0.82	-6.70	-3.48	-6.22	< .001**	-0.29
	PH → MCA	3.61	0.62	2.39	4.84	5.80	< .001**	.32
Indirect	C → MD → MCA	-4.83	0.98	-6.75	-2.90			
	C → PH → MCA	8.72	1.89	5.12	12.55			
Direct	C → MCA	0.59	2.51	-4.34	5.52	0.23	.814	
Total	C → MCA	4.48	2.09	0.37	8.58	2.14	< .05*	0.21

Student Loan Debt								
				95% C.I.				
Type	Effect	Estimate	*SE*	Lower	Upper	*t*	*p*	β
Component	C → MD	1.12	0.09	0.95	1.29	12.68	< .001**	0.96
	C → RA	-0.28	0.09	-0.45	-0.11	-3.29	< .05*	-0.28
	C → PH	3.02	0.12	2.79	3.26	25.40	< .001**	1.47
	MD → MCA	-4.74	0.91	-6.53	-2.95	-5.20	< .001**	-0.27
	RA → MCA	0.16	0.89	-1.60	1.91	0.17	.862	0.01
	PH → MCA	2.50	0.65	1.22	3.78	3.84	< .001**	0.25
Indirect	C → MD → MCA	-5.31	1.23	-7.81	-2.97			
	C → RA → MCA	-0.04	0.27	-0.61	0.47			
	C → PH → MCA	7.55	1.97	3.66	11.47			
Direct	C → MCA	-0.53	2.59	-5.62	4.55	-0.21	.837	
Total	C → MCA	1.66	1.76	-1.80	5.12	-.94	.346	0.08

Brexit								
				95% C.I.				
Type	Effect	Estimate	*SE*	Lower	Upper	*t*	*p*	β
Component	C → MD	1.14	0.10	0.95	1.33	11.78	< .001**	0.90
	C → PH	2.11	0.14	1.84	2.38	15.69	< .001**	1.11
	MD → MCA	-2.97	0.84	-4.61	-1.32	-3.54	< .001**	-0.18
	PH → MCA	3.24	0.60	2.06	4.41	5.42	< .001**	.29
Indirect	C → MD → MCA	-3.37	1.10	-5.53	-1.22			
	C → PH → MCA	6.83	1.52	3.88	9.80			
Direct	C → MCA	-4.92	2.16	-9.16	-0.67	-2.27	< 0.05*	
Total	C → MCA	-1/46	1.80	-4.99	2.07	-0.81	.418	-0.07

Note. C = humorous vs. non-humorous (satirical) messages; MD = message discounting; RA = resource allocation; PH = perceived humor; MCA = message-congruent attitudes. * Significant at the .05 level. ** Significant at the 0.001 level.

Table D4.

Mediation analysis on the relationship between humorous satirical vs. non-humorous (satirical) messages on message-congruent attitudes through emotional and excitative responses.

Climate Change								
				95% C.I.				
Type	Effect	Estimate	*SE*	Lower	Upper	*t*	*p*	β
Component	C → H	0.48	0.10	0.29	0.67	4.93	< .001**	0.46
	C → A	-0.77	0.16	-1.08	-0.46	-4.88	< .001**	-0.46
	C → W	-0.63	0.17	-0.95	-0.30	-3.80	< .001**	-0.36
	C → PH	2.46	0.15	2.16	2.76	16.23	< .001**	1.25
	H → MCA	0.30	0.92	-1.51	2.11	0.33	.743	0.01
	A → MCA	-2.01	0.66	-3.31	-0.72	-3.06	< .05*	-0.14
	W → MCA	7.69	0.63	6.45	8.84	12.12	< .001**	0.57
	PH → MCA	1.67	0.60	0.50	2.85	2.79	< .05*	0.14
Indirect	C → H → MCA	0.14	0.51	-0.91	1.12			
	C → A → MCA	1.55	0.67	0.36	2.99			
	C → W → MCA	-4.82	1.36	-7.50	-2.19			
	C → PH → MCA	4.12	1.74	0.72	7.53			
Direct	C → MCA	-0.95	2.45	-5.76	3.86	-0.39	.699	
Total	C → MCA	0.05	2.26	-4.40	4.49	0.02	.983	0.00

Student Loan Debt								
				95% C.I.				
Type	Effect	Estimate	*SE*	Lower	Upper	*t*	*p*	β
Component	C → H	0.37	0.09	0.19	0.56	3.95	< .001**	0.33
	C → A	-0.44	0.14	-0.72	-0.15	-3.03	< .05*	-0.26
	C → PH	2.77	0.13	2.52	3.03	21.50	< .001**	1.36
	H → MCA	1.87	0.82	0.26	3.47	2.29	< .05*	0.09
	A → MCA	2.72	0.52	1.69	3.74	5.21	< .001**	0.22
	PH → MCA	1.33	0.59	0.16	2.49	2.23	< .05*	0.13
Indirect	C → H → MCA	0.69	0.32	0.13	1.40			
	C → A → MCA	-1.19	0.50	-2.26	-0.35			
	C → PH → MCA	3.68	1.88	0.04	7.49			
Direct	C → MCA	-1.54	2.37	-6.20	3.12	-0.65	.516	
Total	C → MCA	1.64	1.79	-1.88	5.16	0.92	.360	0.08

Brexit								
				95% C.I.				
Type	Effect	Estimate	*SE*	Lower	Upper	*t*	*p*	β
Component	C → H	0.44	0.08	0.27	0.60	5.19	< .001**	0.44
	C → A	-0.33	0.13	-0.59	-0.08	-2.59	< .05*	-0.22
	C → W	-0.43	0.15	-0.71	-0.14	-2.92	< .05*	-0.25
	C → E	0.31	0.08	0.15	0.46	3.79	< .001**	0.32
	C → PH	2.17	0.15	1.88	2.45	14.78	< .001**	1.08
	H → MCA	0.38	1.10	-1.78	2.53	0.34	.731	0.02
	A → MCA	-0.62	0.69	-1.97	0.72	-0.91	.364	-0.05
	W → MCA	2.97	0.61	1.77	4.16	4.88	< .001**	-0.25
	E → MCA	-1.03	1.17	-3.33	1.26	-0.89	.376	-0.05
	PH → MCA	2.39	0.54	1.34	3.44	4.45	< .001**	0.23

Indirect	C → H → MCA	0.16	0.50	-0.80	1.20			
	C → A → MCA	-.21	0.34	-0.38	-.95			
	C → W → MCA	-1.26	0.54	-2.45	-0.35			
	C → E → MCA	-0.32	0.37	-1.07	0.43			
	C → PH → MCA	5.17	1.41	2.44	8.05			
Direct	C → MCA	-6.34	2.03	-10.33	-2.34	-3.12	< .05*	
Total	C → MCA	-2.37	1.77	-5.84	1.10	-1.34	.181	-0.12

Note. C = humorous vs. non-humorous (satirical) messages; H = happiness; A = anger; W = worry; E = excitement; PH = perceived humor; MCA = message-congruent attitudes. * Significant at the .05 level. ** Significant at the 0.001 level.
