# Supplementary material for: The underlying mechanisms of the persuasiveness of different types of satirical news messages
Source: Discourse Process. 2024 Aug 20;61(10):479–97. doi: 10.1080/0163853X.2024.2381407 (PMC11614037; doi:10.1080/0163853X.2024.2381407)
Supplement: Supplemental Material [file HDSP_A_2381407_SM9824.pdf]

Appendix F: Statistic Information ANOVAs

Table F1.

Results of One-Way ANOVAs between all different types of (satirical) messages on dependent variables.

Experiment 1-3		Experiment 1: Climate Change					Experiment 2: Student Loan Debt					Experiment 3: Brexit				
IV	DV	*df*	Error *df*	*F*	*p*	η_p^2	*df*	Error *df*	*F*	*p*	η_p^2	*df*	Error *df*	*F*	*p*	η_p^2
All Different Types of (Satirical) Messages	Message Perceptions															
	Perceived Humor	2	483	138.57	< .001**	0.37	3	540	256.77	< .001**	0.59	3	537	89.56	< .001**	0.33
	Cognitive Responses															
	Message Discounting	2	483	36.37	< .001**	0.13	3	540	59.85	< .001**	0.25	3	537	56.97	<.001**	0.24
	Resource Allocation	2	483	0.17	.846	0.00	3	540	5.89	< .001**	0.03	3	537	0.83	.480	0.01
	Persuasion															
	Message-Congruent Attitudes	2	483	2.23	.109	0.01	3	540	0.76	0.516	0.00	3	537	0.29	.831	0.00

Experiment 4-6		Experiment 4: Climate Change					Experiment 5: Student Loan Debt					Experiment 6: Brexit				
IV	DV	*df*	Error *df*	*F*	*p*	η_p^2	*df*	Error *df*	*F*	*p*	η_p^2	*df*	Error *df*	*F*	*p*	η_p^2
All Different Types of (Satirical) Messages	Message Perceptions															
	Perceived Humor	2	486	131.52	< .001**	0.25	3	539	187.66	< .001**	0.51	3	532	76.23	< .001**	0.30
	Emotional Responses															
	Hopefulness	2	486	1.98	.139	0.01	3	539	0.37	.777	0.00	3	532	0.28	.838	0.00
	Happiness	2	486	12.19	< .001**	0.05	3	539	5.46	< .001**	0.03	3	532	9.00	< .001**	0.05
	Anger	2	486	12.40	< .001**	0.05	3	539	4.96	< .05*	0.03	3	532	2.73	< .05*	0.02
	Worry	2	486	9.43	< .001**	0.04	3	539	1.61	.187	0.01	3	532	3.39	< .05*	0.02
	Excitative Responses															
	Excitement	2	486	1.85	.159	0.01	3	539	1.31	.272	0.01	3	532	4.82	< .05*	0.03
	Persuasion															
	Message-Congruent Attitudes	2	486	0.25	.781	0.00	3	539	1.03	.378	0.01	3	532	1.60	.188	0.01

Note. * Significant at the .05 level. ** Significant at the 0.001 level.
